# Preservation of Cavernosal Erectile Function after Soft Penile Prosthesis Implant in Peyronie's Disease: Long-Term Followup

**DOI:** 10.1155/2008/646052

**Published:** 2008-12-02

**Authors:** Marco Grasso, Caterina Lania, Flavio Fortuna, Salvatore Blanco, Igor Piacentini

**Affiliations:** ^1^Department of Urology, San Gerardo Hospital, 20052 Monza, Italy; ^2^Department of Urology, San Raffaele Hospital, 20132 Milan, Italy; ^3^Department of Urology, Desio Hospital, 20033 Desio, Italy

## Abstract

The aim of this retrospective study is to evaluate the long-term followup of soft penile SSDA prosthesis, without plaque surgery in the treatment of Peyronie's disease. This study included 12 men with Peyronie's disease who underwent placement of a penile prosthesis. All patients were followed for at least 6 years. Prosthesis straightened the penile shaft in all cases, restoring patient sexual satisfaction. No operative or postoperative complications occurred, and no reoperations were needed. All patients have undergone further examination with basal and dynamic eco color Doppler. The findings are encouraging as the penis preserves the ability to enhance the tumescence and penile girth. We can conclude that SSDA penile prosthesis is safe and effective in Peyronie's disease.

## 1. INTRODUCTION

In 1743, de la Peyronie first described the characteristic penile curvature, nowadays known as Peyronie's disease or induratio penis plastica (IPP) [1]. In general, IPP refers to acquired penile deformities during erection (curvature and strictures) and/or penile shortening. This condition is usually associated with palpable hardening (plaque) on the penile shaft and eventually painful erection. Current researches suggest that IPP could be a localized connective tissue disorder affecting the tunica albuginea of the corpora cavernosa.

The aetiology of the disease remains unknown and many hypotheses have been formulated so far. Trauma is thought to be the promoter factor of IPP, causing mechanical stress and microvascular damage. As a consequence, even genetically induced, hyperactive wound healing may cause chronic inflammation and fibrosis of tunica albuginea with subsequent development of hardening and deformation of the penile shaft characteristic of the stabilized phase of disease.

Another hypothesis is related to a defect of immune response causing antibody reaction against the tunica albuginea. IPP can occur in a familiar pattern and it has been reported in association with Dupuytren's disease as well.

Over years, various medical treatments such as radiation, laser therapy, ultrasound, shock wave lithotripsy have been reported without any of them being superior to others. However, surgery is considered the only effective treatment for stabilized disease when severe curvature or narrowing interferes with sexual intercourse [2–4].

Several surgical options have been proposed. Tunica shortening procedures are performed by reducing the length of the convex side of the penis opposite to the penile plaque (Nesbitt or Yachia procedures; plication of corpora cavernosa). Conversely, lengthening techniques are performed by plaque incision or excision while the resulting defect is then covered by a graft. As a result, the extension of the short side of the penile curvature gives the penile shaft the original length [2, 4–6]. Another possible treatment, especially with associated erectile dysfunction, is penile prosthesis placement. These surgical treatments warrant adequate correction of penile curvature and recovery of patient sexual satisfaction [7–9].

We have treated a patient series by placing a particular semirigid soft penile prosthesis (silicon soft dynamic antiextrusion (SSDA)) in which the cavernous tissue is displaced by prosthesis shaft as a peripheral layer of cavernous surrounding tissue.

Our hypothesis was that the residual function of this cavernous tissue could have a positive impact on penile curvature treatment and patient satisfaction. We report our retrospective study with a long-term followup.

## 2. MATERIAL AND METHODS

Between 1998 and 2001, 12 patients (36 to 67 years) (Table 1) were treated by soft penile prosthesis placement (SSDA). All patients presented erectile dysfunction associated with penile deformation. We previously excluded patients with diabetes and vasculogenic impotence. A severe penile shortening was reported in 6 patients. All patients were previously treated by various medical and physical therapies without any benefit. In all patients, the plaque was easily palpable on the penile shaft. Erectile function was tested by duplex dynamic color Doppler ultrasound, nocturnal penile tumescence, and hormone assay.

### 2.1. Description of SSDA prosthesis and surgical procedure

**Figure 1:**
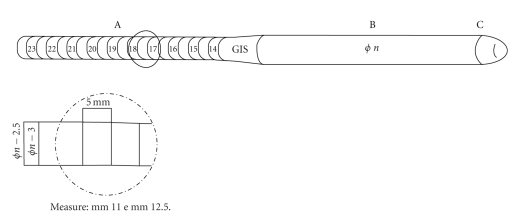
Prosthesis SSDA.

We reviewed our experience with implants on soft penile prosthesis called silicon soft dynamic antiextrusion (SSDA) (Figure 1). This prosthesis consists of silicone elastomer which has three zones with different features:

- *the central zone* has a variable rigidity and size to satisfy different clinical necessities and to permit an easy insertion;
- *the distal zone* is made of softer silicone to reduce the risk of extrusion;
- *the proximal zone* presents a series of slightly cone-shaped segments, with a size of 3 mm, smaller than the central zone, to facilitate the insertion into corpora cavernosa. It requires less dilatation even in the presence of severe fibrosis and can reduce crural pain because of better flexibility [7, 8].

### 2.2. Surgical procedure

A penoscrotal longitudinal incision is our preferred surgical approach for SSDA prosthesis placement. Trichotomy is performed two hours before surgery and short-term antibiotic prophilaxis (Piperacillin 2 g and Netilmicin 150 mg) is administered. The patient is placed in lithotomic position under spinal anaesthesia. We perform a minimal longitudinally corporotomy (<2 cm), on each corpora cavernosa, and then we place the cylinder with appropriate length through the corporotomy. Routinely, a transurethral catheter is left in place until the first postoperative day. Each patient has been followed up until the surgical wound had healed and any surgical complications have been recorded in detail. Patients were then taught to manipulate the penile prosthesis and were allowed to start sexual activity after 6 weeks [10].

### 2.3. Followup

All patients underwent an annual clinical assessment. All patients reached a 6 years minimum followup. At this time, a questionnaire was administered to the patient and his partner. The questions regarded the frequency of sexual intercourse per month, the acceptance degree by patient and his partner (range 0–10), and the overall sexual satisfaction. In order to investigate the postoperative residual function of the corpora cavernosa, all patients were evaluated by color Doppler dynamic ultrasonography (Esaote-Technos, probe 7, 5–10 MHz) before and after taking oral 50 mg Sildenafil associated to visual sex stimulation. We measured thickness of cavernous tissue, peak systolic velocity, and the presence of plaques.

## 3. RESULTS

**Figure 2:**
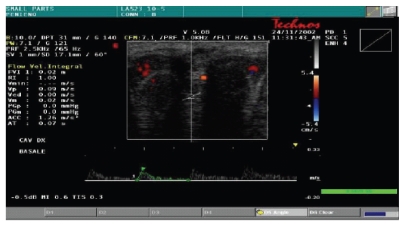
Basal cavernosal flow.

**Figure 3:**
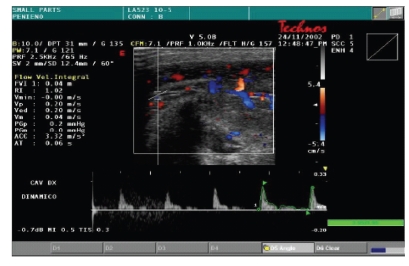
Dynamic cavernosal flow.

**Figure 4:**
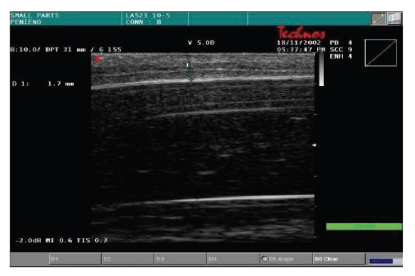
Basal thickness of cavernosal tissue.

**Figure 5:**
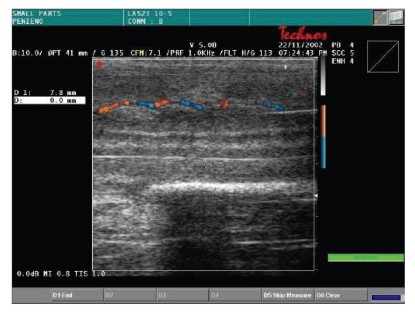
Dynamic thickness of cavernosal tissue.

The implantation of an SSDA prosthesis straightened the penile shaft in all cases indicating a good surgical outcome and restored sexual satisfaction. All the patients have been discharged within the third postoperative day. We did not have any intraoperative complications. Only one patient had a wound infection without permanent consequences. No subsequent postoperative complications were encountered. Only one man reported less than 1 sexual intercourse in a month, 7 men indicated having 1 to 6 sexual intercourses and 4 men more than 6 sexual intercourses (Table 2). The degree of acceptance by couple was 7, 2 (range 4–10) for men and 7, 8 (range 5–9) for the partner. The overall sexual satisfaction was positive in 11 patients (Table 3). The color Doppler dynamic ultrasonography showed a significant thickness increase of cavernosal tissue (5 to 9 mm) as well as peak systolic velocity increase (7.5 cm/s to 16.5 cm/s) after the dynamic phase; no plaques were detected (Table 4) (Figures 2 and 3; Figures 4 and 5). In all cases, we noted an almost complete straightening of the penile shaft.

## 4. DISCUSSION

The natural history of IPP can be summarized into early and late stages. The early stage is characterized by reactive inflammation with multifocal spreading into the tunica albuginea. Clinically, a palpable nodule or plaque that makes penis deformed in its shape during a usually painful erection can be shown. In the second phase, fibrosis and calcification of acute inflammation take place making the plaque hard and steadily causing a stable penile curvature, stricture, and some grade shortening during erection. Reduction of both cavernous blood supply and the possibility of venous leakage due to the rigidity of tunica albuginea may cause some grade of erectile dysfunction.

This histological evolution seems to be constant, while the progression and timing of disease remain unpredictable. We do not have well-defined criteria to establish the end of the process: a quote of patients indeed presents recurrence after long time. Moreover, in younger patients, the course may be more severe [11].

For these reasons, in our opinion the surgical treatment of Peyronie's disease should be as simple as possible, even considering the possible multifocal spreading of fibrosis. Moreover, in order to adhere to patient's perspectives, the ideal prosthetic implant should provide a firm and straightened penis, if possible, restoring the original length or girth of the natural erection [12].

The SSDA soft prosthesis satisfies all these criteria as it provides a good hardening and girth of the penis, implant is easy to perform, it has low cost and low mechanical failure rate while an adequate flexibility warranted by structure, shape, and by intrinsic silicone characteristics lead to a good patient tolerance and comfort. Moreover, after implantation, the residual cavernous surrounding tissue is kept intact with an adequate cavernous arterial blood flow making possible its adequate expansion under sexual stimulation. Furthermore, the characteristic softy tip lowers the pressure of the prosthetic shaft on the tip of corpora cavernosa with possible positive impact on pain and eventually on extrusion rate.

Our technique does not include the plaque treatment associated to prosthesis placement. Even in severe penile curvature, the simple placement of the cylinders makes the penile shaft less pronounced. Interestingly, we noted that over time the same penile curvature decreases until almost disappearing. It can be hypothesized that it may be due to continuous mechanical straighten induced by prosthesis associated with residual cavernous tissue function.

A peculiarity of our study is the dynamic study of residual cavernous tissue that gives the basis for a role of pharmacologic rehabilitative postoperative therapy in order to increase vasoactive response to sexual stimulation and improve patients' satisfaction.

If evaluation of patient satisfaction might be considered adequate considering the long-term followup, an even longer followup would be needed to rule out any long-term mechanical failure as well as spontaneous prosthesis extrusion that did not happen in the present study.

## 5. CONCLUSION

Silicon soft dynamic antiextrusion penile prosthesis is safe and effective in the treatment of severe Peyronie's disease associated to penile deformity during erection. Results in terms of penile curvature correction are good. The majority of patients report a sexual satisfaction. Moreover, the positive response to vasoactive drugs by residual cavernous tissue might give the rationale for a pharmacological adjuvant rehabilitation therapy in order to improve the patient satisfaction even in patients having an insufficient residual cavernous tissue erection.

## Figures and Tables

**Table 1:** 

Age	No. of patients
30–40	1
40–50	3
50–60	7
60–70	1

Total	12

**Table 2:** 

No. of sexual intercourses in a month	No. of patients
Less than 1	1
Between 1 and 6	7
More than 6	4

**Table 3:** 

	Mean	Range
Acceptance degree for men (1–10)	7, 2	4–10
Acceptance degree for partner (1–10)	7, 8	5–9
Overall satisfaction (yes-no)	Yes 11 pts (91%)	No 1 pt (9%)

**Table 4:** 

	Basal		Dynamic	
	*Range*	*Mean*	*Range*	*Mean*
Thickness of cavernosal tissue	1,7–2,2 mm	1,9 mm	2,8–7,2 mm	5 mm
Peak systolic value	6–9 cm/sec	7,5 cm/sec	13–20 cm/sec	16,5 cm/sec

## References

[1] de la Peyronie FG (1743). Sur quelques obstacles qui s'opposent à l'éjaculation naturelle de la semence. *Memoires de I'Academic Royale des Sciences Montpellier Chir*.

[2] Garber BB (1996). Inflatable penile prosthesis: results of 150 cases. *British Journal of Urology*.

[3] Grasso M, Lania C, Ronchi F, Colombo R, Di Girolamo V, Rigatti P A report of our experience in penile prosthesis implant.

[4] Grasso M, Ronchi F, Di Girolamo V Our experience in penile prosthesis implant.

[5] Shabsigh R (1998). Penile prostheses toward the end of the millenium. *The Journal of Urology*.

[6] Chiang H-S, Wu C-C, Wen T-C (2000). 10 years of experience with penile prosthesis implantation in Taiwanese patients. *The Journal of Urology*.

[7] Burns-Cox N, Burston A, Gingell JC (1997). Fifteen years experience of penile prosthesis insertion. *International Journal of Impotence Research*.

[8] Evans C (1998). The use of penile prostheses in the treatment of impotence. *British Journal of Urology*.

[9] Rogers E, Murphy DM (1997). Use of the AMS inflatable penile prosthesis in the management of erectile impotence. *Irish Medical Journal*.

[10] Lania C, Grasso M, Franzoso F, Blanco S, Rigatti P (2004). Peyronie's disease, natural history. *The Journal of Urology*.

[11] Chew KK, Stuckey BGA (2000). Use of transurethral alprostadil (MUSE) (prostaglandin E1) for glans tumescence in a patient with penile prosthesis. *International Journal of Impotence Research*.

[12] Mireku-Boateng AO, Dennery M, Littlejohn J (2001). Use of Sildenafil in penile implant patients. *Urologia Internationalis*.

